# Venn-diaNet : venn diagram based network propagation analysis framework for comparing multiple biological experiments

**DOI:** 10.1186/s12859-019-3302-7

**Published:** 2019-12-27

**Authors:** Benjamin Hur, Dongwon Kang, Sangseon Lee, Ji Hwan Moon, Gung Lee, Sun Kim

**Affiliations:** 1 Interdisciplinary Program in Bioinformatics, Seoul National University, 1 Gwanak-ro, Seoul, Korea; 2 Department of Computer Science and Engineering, 1 Gwanak-ro, Seoul, Korea; 3 Bioinformatics Institute, Seoul National University, 1 Gwanak-ro, Seoul, Korea; 4 National Creative Research Initiatives Center for Adipose Tissue Remodeling, Institute of Molecular Biology and Genetics, Department of Biological Sciences, Seoul National University, 1 Gwanak-ro, Seoul, Korea

**Keywords:** Venn diagram, Differentially expressed genes, Network propagation, Gene prioritization

## Abstract

**Background:**

The main research topic in this paper is how to compare multiple biological experiments using transcriptome data, where each experiment is measured and designed to compare control and treated samples. Comparison of multiple biological experiments is usually performed in terms of the number of DEGs in an arbitrary combination of biological experiments. This process is usually facilitated with Venn diagram but there are several issues when Venn diagram is used to compare and analyze multiple experiments in terms of DEGs. First, current Venn diagram tools do not provide systematic analysis to prioritize genes. Because that current tools generally do not fully focus to prioritize genes, genes that are located in the segments in the Venn diagram (especially, intersection) is usually difficult to rank. Second, elucidating the phenotypic difference only with the lists of DEGs and expression values is challenging when the experimental designs have the combination of treatments. Experiment designs that aim to find the synergistic effect of the combination of treatments are very difficult to find without an informative system.

**Results:**

We introduce Venn-diaNet, a Venn diagram based analysis framework that uses network propagation upon protein-protein interaction network to prioritizes genes from experiments that have multiple DEG lists. We suggest that the two issues can be effectively handled by ranking or prioritizing genes with segments of a Venn diagram. The user can easily compare multiple DEG lists with gene rankings, which is easy to understand and also can be coupled with additional analysis for their purposes. Our system provides a web-based interface to select seed genes in any of areas in a Venn diagram and then perform network propagation analysis to measure the influence of the selected seed genes in terms of ranked list of DEGs.

**Conclusions:**

We suggest that our system can logically guide to select seed genes without additional prior knowledge that makes us free from the seed selection of network propagation issues. We showed that Venn-diaNet can reproduce the research findings reported in the original papers that have experiments that compare two, three and eight experiments. Venn-diaNet is freely available at: http://biohealth.snu.ac.kr/software/venndianet

## Introduction

A biological experiment is generally designed to characterize the biological mechanism underlying different phenotypes. Transcriptome, or gene expression profile in the whole cell, provides a holistic picture of a cell at the fine-grained level, individual gene. In transcriptome studies, identifying differentially expressed genes (DEGs) is the first step to understand the difference between control and treated samples in transcriptome level. Some experiment designs have multiple lists of DEGs to address complicated biological questions that tries to narrow down the subset of genes. When the lists of DEGs increases, summarizing the relationship between lists becomes much more challenging. Therefore, Venn diagram, an effective method that can effectively summarize and illustrate the portion of each gene sets is generally used. Venn diagram is an intuitive interpreter that helps researchers to understand common or distinctive characteristics of the experiments and helps researchers make the decision for further investigation. However, there are several issues when Venn diagram is used to compare and analyze experiments that have multiple DEG lists.

First, current Venn diagram tools do not offer enough information to identify genes that are related to the phenotype differences. Most of the current Venn diagram tools are developed to visualize a Venn diagram in a easy-to-understand or assists researchers’ understanding of the experiment in terms of the number of genes in each section of a Venn diagram [1–9]. These tools are very useful but they do not give effective method to design an effective follow-up study to further investigate which genes are more related to the phenotype differences in multiple biological experiments. For example, in some scenario, the researcher might be interested to focus on DEGs that satisfies three experimental conditions (in other words, the intersection of three distinct DEG lists). However, the number of genes in the following condition might have too many genes to be considered and it is generally difficult to prioritize the most promising genes because the candidate DEGs have three distinct ranks that are corresponded to each DEG lists.

Second, prioritizing genes that satisfy the researcher’s interests only with the lists of DEGs and expression values are challenging when the experiment is designed to have the combination of treatments. For instance, when researchers designed an experiment to investigate the synergy effect of two different treatments (for convenience: drug A, drug B), the experiment will have three lists of DEGs to compare: drug A, drug B, and drug A+B, respectively. The relative complement of DEGs from the combination of drug A and drug B, can logically represents the synergy effect of two different treatments, but it cannot illustrate which drug had more efficacy to the expression alteration. Also, DEGs from the intersection of three different lists cannot be ignored. Some DEGs might have been boosted by the combination of drugs. However, these DEGs might be underestimated because these genes are also differentially expressed in other treatments and it is difficult to create the decision criteria of how whether the expression alteration of the drug combination is outstanding than others.

In this paper, we show that the two issues can be effectively handled by ranking or prioritizing genes in regions of a Venn diagram. Thus, a gene prioritization strategy needs to be implemented into the Venn diagram in order to rank the DEGs of each region. Gene prioritization is a widely used strategy that rank genes by combining multiple data sources (including methods) to maximize the biological relevance to answer difficult questions that cannot be easily solved in a single data. Among various strategies of prioritizing genes, network propagation is one of the widely used technique that computes the influence of initial nodes (or seeds) to other nodes [10], and can prioritize genes in the context of biological networks [11–17]. However, selection of seed genes is one of the critical factors for the network propagation and becomes more important when prior knowledge is not available or is not enough. In this paper, we suggest that the seed selection issue can be handled by allowing the user to select seed genes with the combinations of regions in a Venn diagram. We argue that each area of the Venn diagram represents a subset of DEGs and each area represents genes that contain biological meaning. And these subsets can be used as a guidance to logically select seeds for the subset of genes that the user is in interested.

Here, we present Venn-diaNet: a web-based Venn diagram based network analysis framework that can prioritize genes to compare multiple biological experiments of transcriptome data. A convenient web-based user interface is provided to generate Venn diagrams of DEGs dynamically and to perform network propagation upon protein-protein interaction (PPI) network to investigate which genes are relevant to certain phenotypes. We believe that Venn diagram, coupled with analytic methods such as network propagation, can be a very useful tool for comparing multiple biological experiments that have multiple different controls.

## Methods

**Fig. 1:**
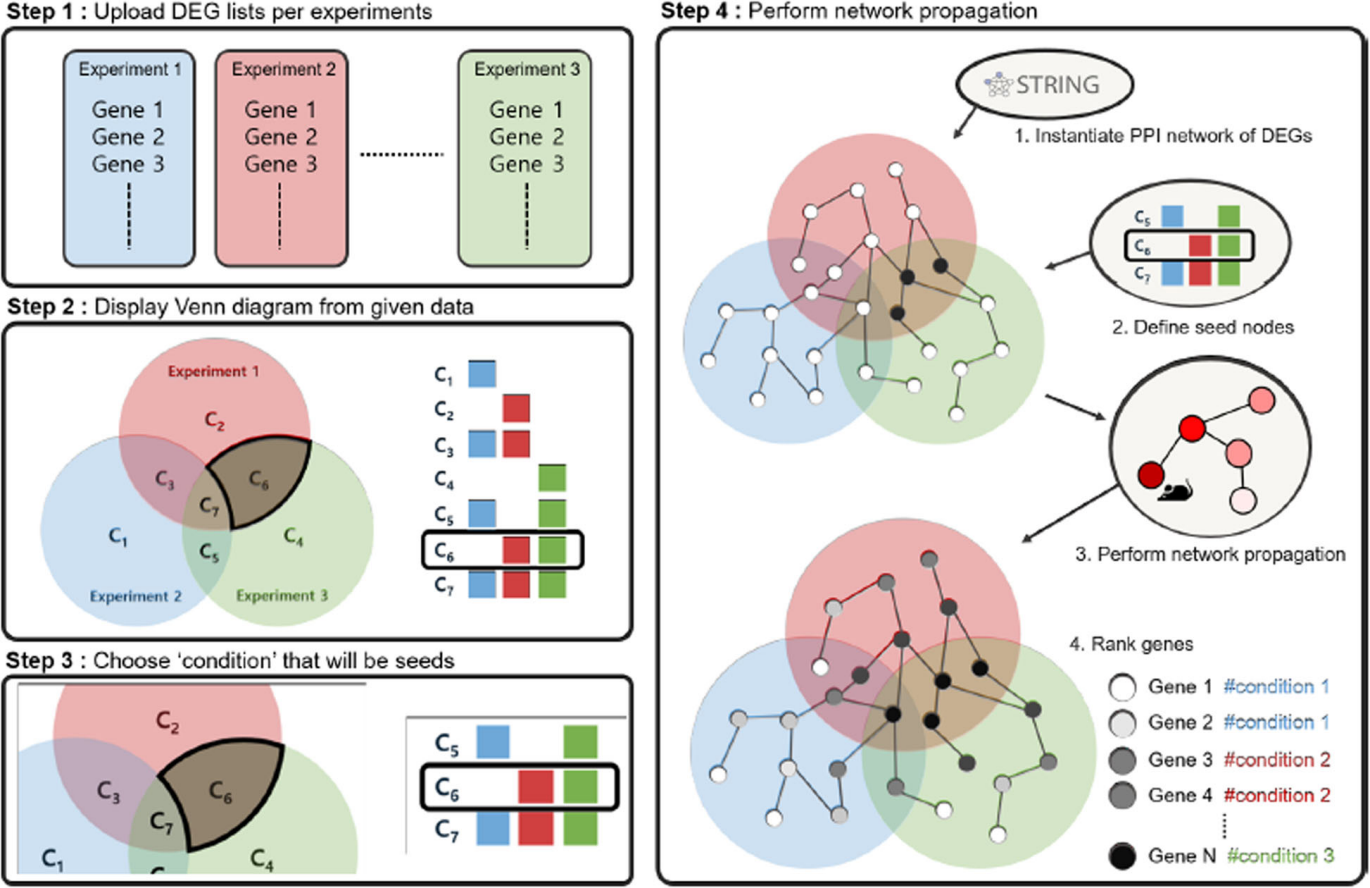
Venn-diaNet work flow. Step 1 : Venn-diaNet, as input, takes DEG lists per experiments from user. Step 2 : Uploaded DEGs from Step 1 are interpreted with a Venn diagram and they are organized as sets with table. Now, a user can repeat Steps 3 and 4 as many times as she wants. Step 3 : One or multiple regions of Venn diagram, C _*i*_, is selected as seeds for further network propagation analysis. Step 4 : Once seed is defined, Venn-diaNet instantiates a PPI network of DEGs from STRING DB. Network propagation with given seeds from the previous steps. As a result, DEGs are ranked by the probability score calculated during the Markov Random Walk

In this section, we explain how Venn-diaNet performs network analysis to prioritize genes from DEGs with Venn diagram and the network propagation technique. The overview is shown in Fig. 1.

### Venn-diaNet work flow

#### STEP 1: Taking input data

Venn-diaNet takes multiple DEG lists as input. Each DEG list is determined by comparing treatment/control or treatment/treatment in the experiment (Fig. 1: Step 1). Each file must include a DEG list from an experiment. For example, if a researcher wants to compare three different experiments, three independent files of DEG list must be provided. The format of the file is as follows. Each input file requires transcript ID (or gene ID) for the first column and gene symbol for the second column. Venn-diaNet takes transcript ID to handle inputs that might annotate identical genes (causing duplicated genes). Currently, Venn-diaNet requires this column but the information does not need to be strict to certain annotation format. We provide an example data on the web page of Venn-diaNet for better understanding.

#### STEP 2: Generating Venn diagram of DEG sets

Venn-diaNet considers each experiment as a set for the diagram. Therefore, With given number (=*n*) of experiments *E*, Venn-diaNet generates a diagram of *n* circles that have a maximum of 2^*n*^−1 regions. Each region is denoted as *C*_*i*_(1≤*i*≤2^*n*^−1) while each *C*_*i*_ contains genes of:

$$ {C}_{i}= \left\{ {g} : {g} \in \bigcap_{j=1}^{N} {G} ({b}_{j}) \right\}   $$

$$ G({b}_{j}) = \left\{\begin{array}{cc} E_{j} & if \, j = 1 \\ E^{c}_{j} & if \, j = 0\\ \end{array} \right.   $$

*b* represents a binary number of *C*_*i*_ (i.e. *C*_1_ = 001) while *b*_*j*_ indicates the position of digits (i.e. *b*_1_ = 1, *b*_2_ = 0, *b*_3_ = 0). If Venn-diaNet receives DEG lists from 3 experiments, Venn-diaNet illustrates a Venn diagram of 3 sets (*E*_1_,*E*_2_,*E*_3_) that have 7 regions (*C*_1_,*C*_2_,*C*_3_, ⋯*C*_7_), where *C*_7_ contains genes of *E*_1_∩*E*_2_∩*E*_3_. We emphasize that *C*_*i*_ represents DEGs that is specific to the corresponding region that could be considered as ‘condition-specific genes’.

#### STEP 3: Seed selection

This step is the most important part of Venn-diaNet. A user can select one or more segments of the Venn diagram (*C*_*i*_) as seeds for network propagation to measure the global influence of the seed DEGs. Thus, the results will vary depending on the selected seeds. As we previously mentioned, network propagation methods generally use informative genes (i.e. ‘disease-related genes’, ‘phenotype-related genes’) as seeds. The idea of network propagation in Venn-diaNet is similar. Since DEGs in each region of the Venn diagram can be considered as condition-specific DEGs, DEGs in *C*_*i*_ can be a guide to find similarities or dissimilarities to other *C*_*j*_(*j*≠*i*) that we are interested in. Because the selection is crucial, we describe three possible seed selection scenarios with examples to help understand the seed selection. Each seed selection scenario describes that user can select seeds from one or more segments from Venn diagram and prioritize genes with specific prospects.

**Fig. 2:**
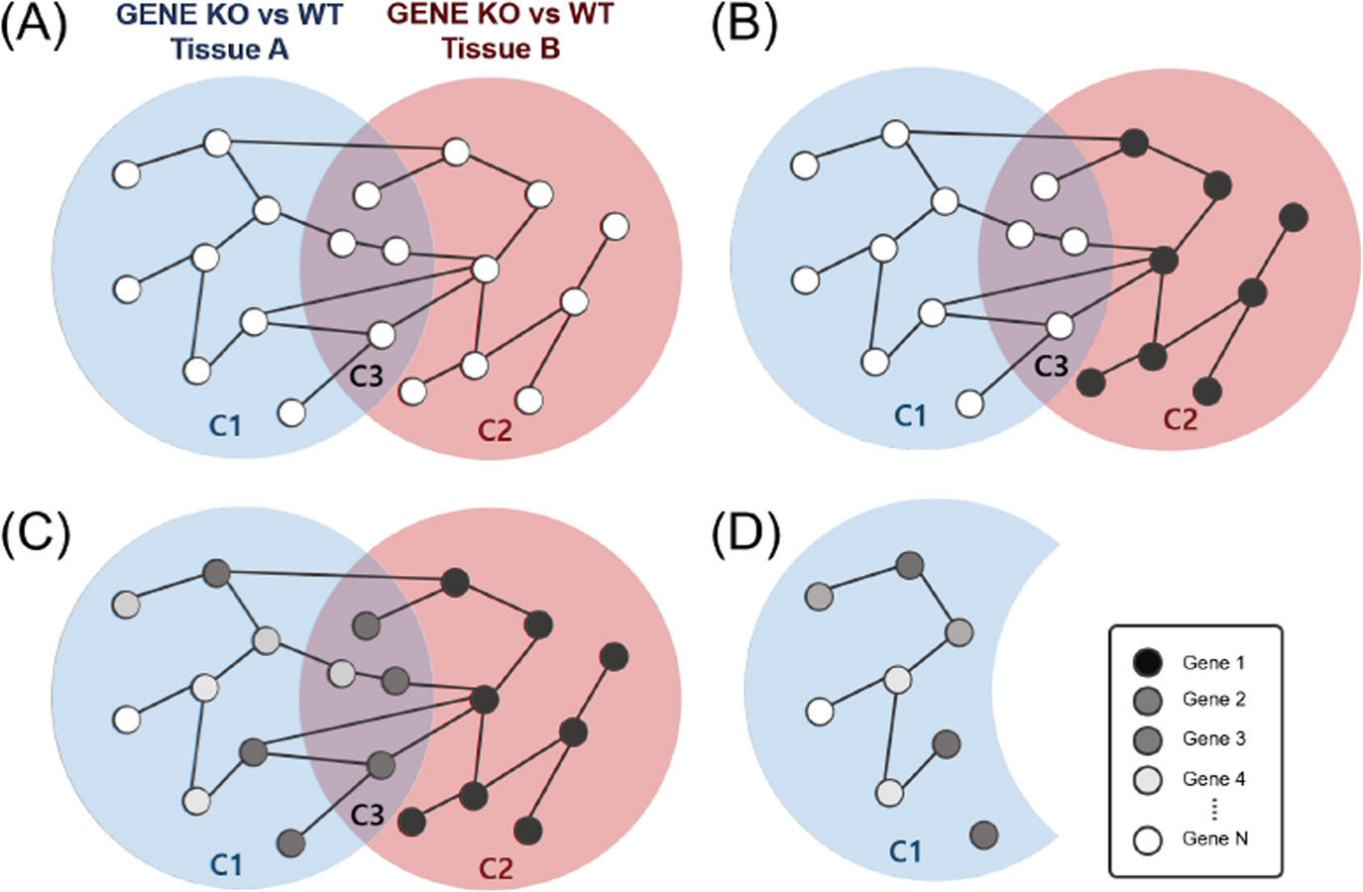
Key concept of Venn-diaNet. **a** Instantiate a PPI network with the DEGs from the multiple experiments. **b** When we are interested in C_1_ that has similar function as C_2_, we can define C_2_ as seeds. **c** Performing network propagation with Markov Random Walk. **d** Discard C_3_ genes (as well as seed genes) in order to focus on C_1_ genes. Remaining genes are ranked by the probability score calculated from the previous step

The first scenario is to consider *‘condition-specific function’* as seeds. Again, DEGs in specific region can be considered as condition-specific DEGs. If we use these genes as seeds, then we can prioritize DEGs belonging to other conditions in terms of functional similarity to the seed DEGs. For example, if a user wants to prioritize tissue A-specific DEGs (Fig. 2A: *C*_1_) that have similar function to the tissue B-specific DEGs when the same genes is knockout (KO), tissue B specific-DEGs (Fig. 2A: *C*_2_) can be used as seeds.

The second scenario is to consider *‘common function’* as seeds. In some cases, a user might be interested in condition specific DEGs that have common function in different experiments. For instance, if the user is interested in tissue A-specific DEGs (Fig. 2A: *C*_1_) that have similar function between two different tissues, *C*_3_ can be seeds. Similarly, if the common KO effect in different tissues are in interest (*C*_3_), *C*_1_ + *C*_2_ can be seeds.

The last scenario suggests to use GO terms to consider seeds that have *‘Functional similarity’* between segments in Venn diagram. This scenario assumes the case that there is no sufficient knowledge to have confidence selecting certain *C*_*i*_ as seeds. This scenario is for users who expect the DEGs of interest (*C*_*j*_) to have functional similarity to the DEGs in other condition (*C*_*i*_) but not certain which *C*_*i*_ is closer to *C*_*j*_. In this case, we suggest using GO terms to compare the similarity between *C*_*i*_ and *C*_*j*_, and choose *C*_*j*_ similarity to the condition of interest will be appropriate to be as seeds. This scenario is suggested as a ‘minimum guideline’ to analyze the data that might not be covered by the fore-mentioned scenarios. Currently, Venn-diaNet does not support GO term analysis, thus the GO term analysis should be conducted separately by the researcher.

#### STEP 4: Network propagation and gene ranking

When a set of seed DEGs are selected, Venn-diaNet instantiates a PPI network of DEGs from STRING DB [18]. In the instantiated network, nodes are DEGs and an edge between two DEGs is defined when the corresponding edge in the original PPI network is of high-confidence (combined score >700). Then, Markov Random Walk (MRW) [19] is performed using the seeds selected in the previous step (Fig. 1: Step 4). The goal of network propagation is to quantify the influence of seed DEGs to the remaining DEGs. The selected seed DEGs can be considered as the hypothesis that a user wants to test. Thus, by performing a network propagation analysis, the user can obtain the DEGs pertaining to the hypothesis. For the network propagation, an R package diffusr, the implementation of MRW, is used. The equation is shown below:

$$ p^{t+1}=(1-r)A^{\prime}p^{t}+rp^{0}   $$

where *p*^0^ is the vector of initialized nodes, *t* is a time step, *p*^*t*^ is the vector at the current time step *t*, *p*^*t*+1^ is the vector at the next time step, *A*^′^ is column-normalized matrix of adjacency matrix *A*, and *r* is the restart rate. *p*^0^ is initialized in 1 or 0, to represent the assigned seed DEGs and target DEGs, and normalized so the sum of the elements in *p*^0^ becomes 1. The adjacency matrix *A* is a matrix consists with 0 or 1 that represents a graph with no weighted edges. The network propagation was performed with the default options of the diffusr package, where *r* is 0.5 and stops the network propagation when *L*1 norm difference between *p*^*t*^ and *p*^*t*+1^ is smaller than 10^−4^. When the algorithm stops, Venn-diaNet returns a ranked gene sets based on the network propagation result.

### Web interface

**Fig. 3:**
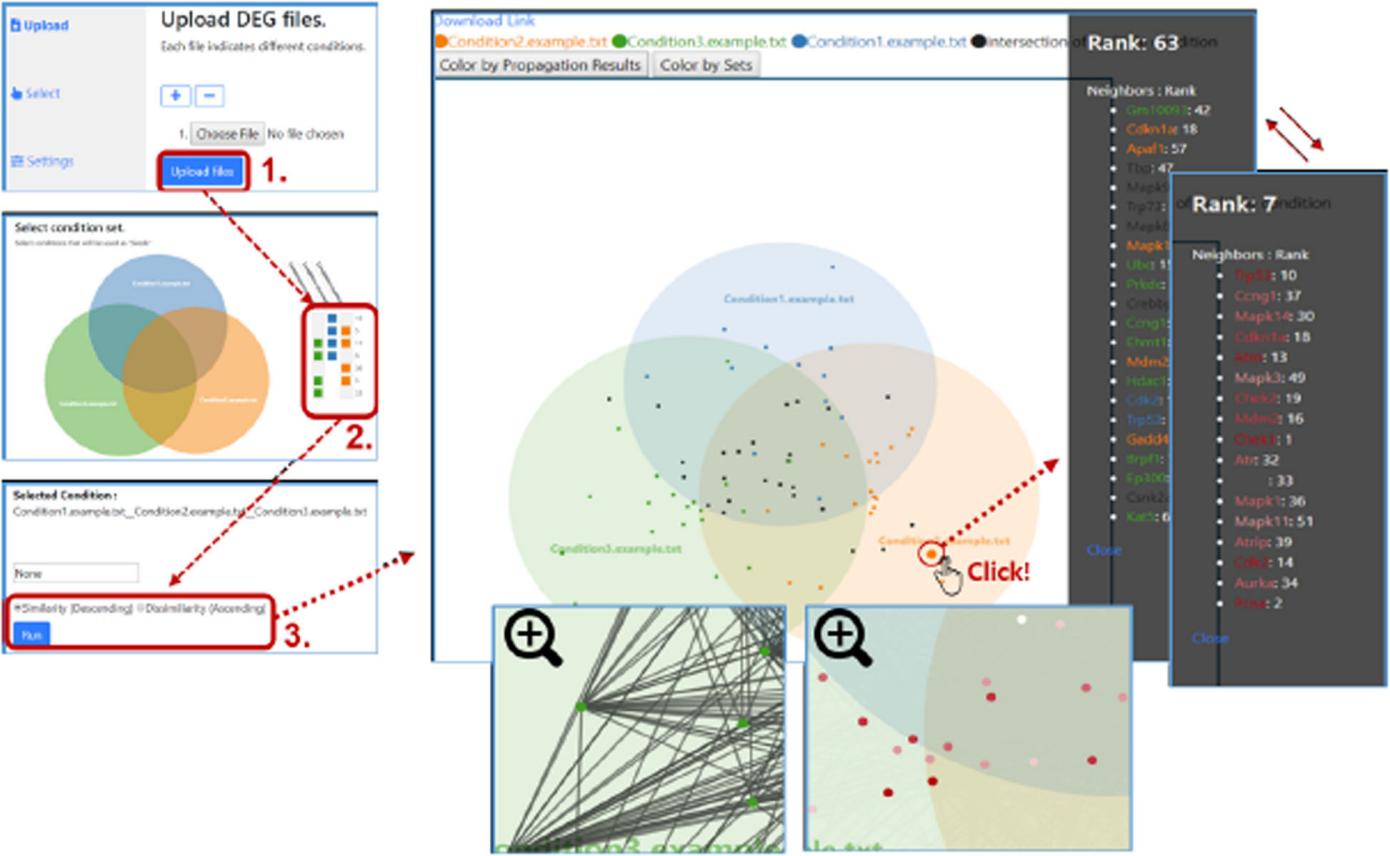
Venn-diaNet (web) work flow. A work flow of Venn-diaNet (web). Step 1: Upload DEG list per experiment. Step 2: Select seed condition *C*_*i*_ Step 3: Perform analysis. Venn-diaNet gives user (1) list of ranked genes, (2) gene’s neighbor nodes information (when the node is clicked). (3) Venn diagram with PPI network (when the Venn diagram is zoomed in)

The web tool of Venn-diaNet’s work flow is summarized in Fig. 3. The details of Venn-diaNet work flow (web) is described in the manual of Venn-diaNet (Additional file 1).

## Results

We evaluated the performance of Venn-diaNet using three datasets downloaded from Gene Expression Omnibus (GEO) [20] or from the supplementary data of the corresponding published paper. The selected dataset is used to determine whether Venn-diaNet can be used in various experimental designs.

### Case 1: Venn-diaNet for two experiments

In order to validate the Venn-diaNet performance for experiment designs that have two experiments, we used a dataset from a study of Per2 KO mice with two different tissues [21]: (*i*) Per2 KO vs Wild type (WT) in white adipose tissue (WAT Per2 KO), and (*ii*) Per2 KO vs WT in brown adipose tissue (BAT Per2 KO). The authors used these two DEGs lists and reported that several WAT specific expressed genes have similar behavior in BAT when Per2 is KO. Two independent DEG lists, BAT Per2 KO and WAT Per2 KO are downloaded from the supported supplementary data.

**Fig. 4:**
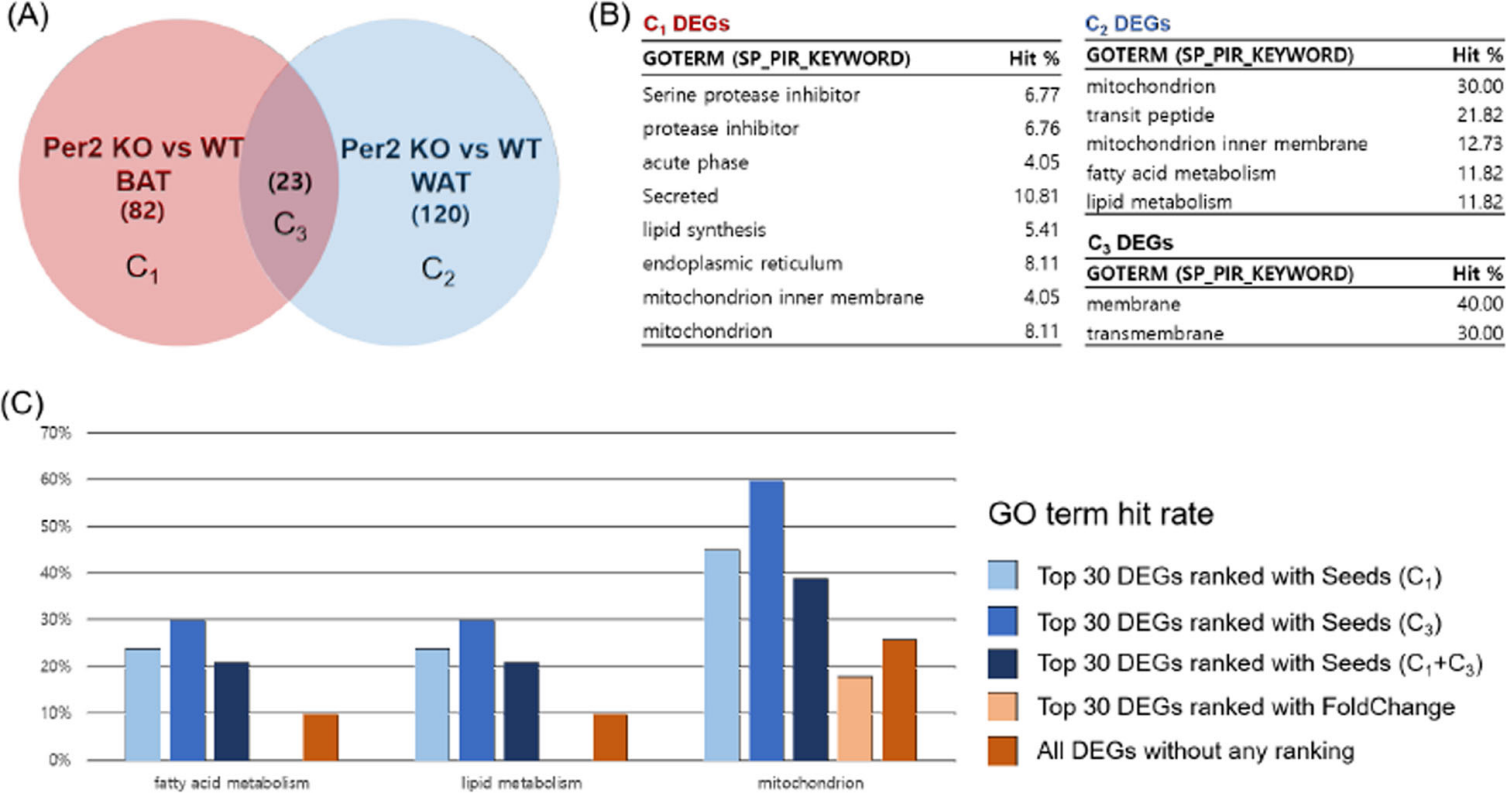
Venn-diaNet Per2 GO term comparison. **a** Venn-diagram of Per2 KO experiment perform by Benedetto Grimaldi et al. *C*_1_ represents Per2 KO vs WT DEGs that is specific to BAT while *C*_2_ represents WAT specific Per2 KO vs WT DEGs. **b** Enriched GO terms by DAVID gene functional clustering analysis. Gene functional clustering was performed for each specific condition (*C*_*i*_). **c** Enriched GO terms of Top 30 genes prioritized by corresponding seeds

For convenience, we will denote BAT Per2 KO specific DEGs as *C*_1_, WAT Per2 KO as *C*_2_, and the intersection DEGs of BAT Per2 KO and WAT Per2 KO as *C*_3_ (Fig. 4A). We used this data to show that Venn-diaNet can reproduce the authors’ results by following the authors inputs, interest, and approach. As we mentioned, the study reported that Per2 KO caused BAT specific genes to express in WAT by controlling PPAR *γ*-dependent genes. Therefore, we set our aim to find promising *C*_2_ DEGs that have the similar characteristic in BAT tissue. For this study, we could use all three suggested seed scenarios to address the authors interest. For each seed scenarios, we compared (*i*) how the GO terms of ranked top 10% genes matches the GO terms reported in the original paper, and (*ii*) how many genes matches to the genes that are reported in the original paper. Note that the authors used only fold change to rank genes and did not use any gene prioritization method.

#### Condition specific function (*C*_1_) & common function (*C*_3_) as seeds

BAT Per2 KO specific DEGs (*C*_1_), can be used as seeds in order to prioritize genes of WAT Per2 KO specific DEGs (*C*_2_). This scenario is to investigate the unknown genes that expresses exclusively in BAT somehow seems to be expressed in WAT when Per2 is KO. The phenomenon indicates that there might be functional similarity between these two different conditions.

Similarly, common DEGs between two experiments (*C*_3_) can also be considered as seeds. The activation of BAT-specific genes in WAT also means that BAT and WAT share common functions. Thus, the common function of these genes (*C*_3_) might be guideline to prioritize WAT specific genes (*C*_2_) with the context of ‘functional similarity’ between two different tissues. It is interesting that Venn-diaNet could prioritize genes in top 30 (about 10% of total candidates) as well as prioritizing genes that are related to the functions that the authors reported (Fig. 4C, Additional file 2:(2)).

#### Analysis scenario with functional similarity (*C*_1_) as seeds

As we discussed in the previous section, we might encounter a situation where the user does not have sufficient knowledge to select seeds. Therefore, we assumed ourselves that we do not have confidence to choose certain seed scenario. In this case, we suggested a ‘minimum guideline’ to choose certain condition as seeds to rank genes in condition of interest. For this, we define it as ‘The condition that have functional similarity to the condition of interest will be appropriate to be as seeds’, which the ‘function of the condition’ can be determined by gene function clustering by DAVID [22, 23].

The process is very straight-forward. (*i*) Find the major GO terms of each *C*_*i*_, (*ii*) use the genes in *C*_*i*_ if the GO terms are similar to the condition *C*_*j*_(*j*≠*i*) that we want to prioritize. As a result, we found that GO term (mitochondrion) in *C*_1_ was similar to the condition of interest (*C*_2_) (Fig. 4B). Thus, *C*_1_ becomes appropriate seed for this scenario and the results shares the same which we discussed in the previous subsection.

Venn-diaNet is also tested with other possible seed scenario (*C*_1_+*C*_3_) to confirm whether Venn-diaNet performs better than random seeds. Genes lists and GO terms that we compared are described with details in (Additional file 2).

### Case 2: Venn-diaNet for three experiments

**Fig. 5:**
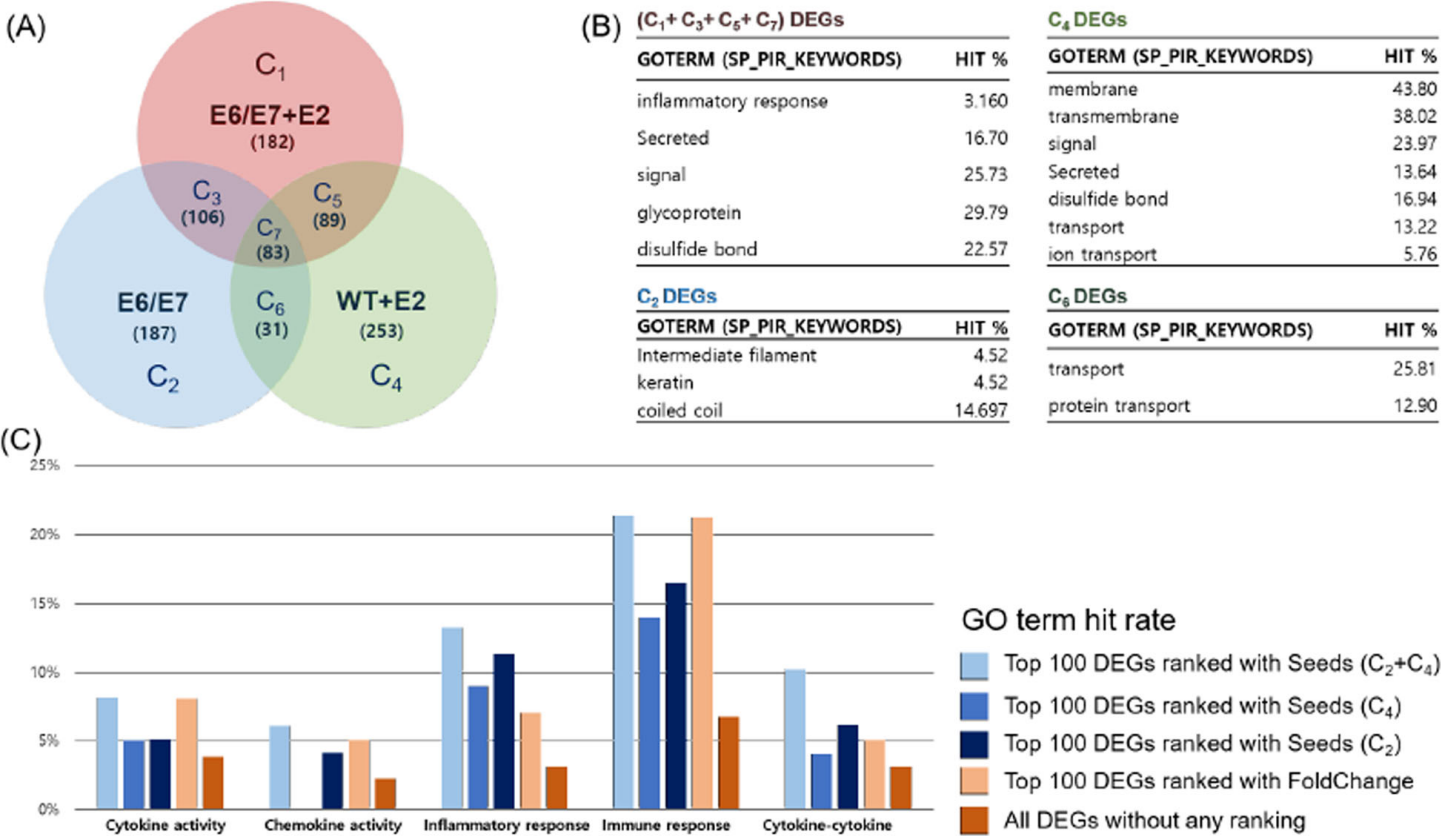
Venn-diaNet HPV experiment GO term comparison. **a** Venn-diagram of E6/E7 experiment performed by Megan E. Spurgeon et al. *C*_1_, *C*_2_, and *C*_4_ represents E6/E7+E2 specific DEGs, E6/E7 specific DEGs, and E2 specific DEGs, respectively. **b** Enriched GO terms by DAVID gene functional clustering analysis. Gene functional clustering was performed for each specific condition (*C*_*i*_). **c** Enriched GO terms of Top 100 genes prioritized by corresponding seeds

Data from a study of human papillomavirus oncogenes [24] is used for Venn-diaNet validation to consider the case of more complicated experiment designs. The study observes the independent, synergistic effects of two treatments: (*i*) K14E6/E7 bitransgenic mice vs WT mice (E6/E7), (*ii*) estrogen treated mice vs WT mice (E2), and (*iii*) K14E6/E7 bitransgenic mice treated with estrogen mice vs WT mice (E6/E7+E2) (Fig. 5).

As the authors did, we focused on E6E7+E2 DEGs (*C*_1_ + *C*_3_ + *C*_5_ + *C*_7_) to determine the synergistic effect of E6/E7 and E2. We selected E6/E7 specific DEGs and E2 specific DEGs (*C*_2_ + *C*_4_) for the seed scenario of ‘condition specific function’. The seed scenario represents that the independent effect of each treatment as a guideline to find the effect of the combined factors. Our goal for this study is to reproduce GO terms and genes that the authors reported. For this study we focused on the results of top 100 genes (from 461 genes), prioritized by Venn-diaNet.

#### Condition specific function as seeds (*C*_2_ + *C*_4_)

We found that Venn-diaNet could prioritize genes and GO terms that are reported in the original paper by using the combination of independent effects of two factors as seeds (*C*_2_ + *C*_4_) (Fig. 5C and Additional file 3:(2)). However, several careful consideration remains to be discussed. When we consider the prioritized top 20% genes, Venn-diaNet was not superior than the original paper’s results, but it could still prioritize genes that are related to the GO terms were the original paper focused. In addition, Venn-diaNet could prioritize other genes that are related to the function of interest (immune response & inflammatory response) that are responsible to the HPV associated cervical cancer while the authors did not.

For example, Tlr2, a gene that is known to be related to take a significant role in HPV associated cervical cancer [25–28], was also over expressed exclusively in E6/E7+E2. The results supports that Tlr2 might also be one of the significant gene that is enhanced by the combined effect of E6/E7 and E2, which achieves the condition of ‘inflammatory response are increased by epithelial E6/E7 expression and further enhanced by estrogen’. We conjecture that Tlr2 was not included in the original paper because the fold change of Tlr2 is not significant (ranked 332^th^ in terms of fold change rankings) enough and become out-focused. However, our gene prioritization analysis ranked Tlr2 much higher in the 33^rd^ place.

Likewise, CD74 is reported that it may play an important role in the pathogenesis and angiogenesis of cervical cancer [29] as well as the influence of the HPV [30]. Venn-diaNet placed this gene in the 76^th^ position while fold change could only rank them as 182^th^. Icam1 was ranked 76^th^ in foldchange but had the 3^rd^ position in Venn-diaNet which also might have a E6/E7+E2 specific expression while Icam1 was also reported to have a role with HPV related cervical carcinoma [31]. The comparison of Top 100 ranked genes related to ‘inflammatory response’ & ‘immune response’ is summarized in Additional file 3:(3).

#### Functional similarity as seeds (*C*_4_)

*C*_4_ was selected by following the ‘minimum guideline’ to select seeds. Unlike ‘Condition specific function as seeds’, seeds chosen by functional similarity performed weaker (Both in GO terms and rankings) than the previous seeds (Fig. 5C and Additional file 3:(3)) This is probably because the seed scenario does not reflect the effect of E6/E7. E6/E7 is well known to change the activity of cytokine and chemokine, and Venn-diaNet could not prioritize those genes without considering those effects in seeds. We would like to emphasize that this seed scenario reflects that using seed genes from a singular treatment is not effective to rank genes that is under the influence of multiple treatments. However, Venn-diaNet could still prioritize 7 genes in top 100 with seeds of ‘functional similarity’ (Additional file 3:(2)). Other possible seeds were also tested and the results indicates other seeds are less effective than the suggested seed scenarios.

### Case 3: Venn-diaNet for eight experiments

Case 3 is a dataset from a study that designed the experiments with four treatments in four tissues [32]: (*i*) narciclasine (ncls), (*ii*) vehicle (veh), (*iii*) high-fat diet (HFD), (*iv*) normal chow diet (NCD), (*v*) WAT, (*vi*) BAT, (*vii*) liver, and (*viii*) muscle. The initial number of sets of this study were extremely complicated that makes almost impossible to interpret the DEG list at once. Thus, the authors used a step-by-step filtering method to find promising genes for these multi-condition data. The authors searched the relation between treatments and tissues using hierarchical clustering and narrowed down to compare two DEG lists (HFD-ncls/HFD-veh: DEGs from the comparison of HFD mice treated with ncls and HFD mice treated with veh, NCD-veh/HFD-veh: DEGs from the comparison of NCD mice treated with veh and HFD mice treated with veh) of muscle. The study reported genes that have low expression level in HFD, changed to have a high expression level when ncls was given. The results indicate that a natural compound ncls can attenuate diet-induced obesity and the associated genes can enhance the energy expenditure.

To reproduce the results of the original paper, we planned two different scenarios. The first scenario is to follow the story of the authors: using two DEG lists. The authors compared the expression profile of treatments and tissues using hierarchical clustering as a very first step. They discovered that muscle had partial mutual exclusive expression pattern to other tissues, and made a hypothesis of ‘ncls might accelerate genes to be expressed again while the genes were suppressed in HFD environment in muscle’. We assumed that we also reached to this step and use Venn-diaNet for the DEGs of HFD-ncls/HFD-veh and NCD-veh/HFD-veh. Venn-diaNet will mimic this story with the concept of ‘Case 1: Venn-diaNet for two experiments’ analysis of Venn-diaNet.

Another scenario is to find promising genes purely by Venn-diaNet, using eight DEG lists. The goal of this scenario is to check whether Venn-diaNet can track down the reported genes, with a reasonable story. We would like to emphasize that the original paper has (i) filtered out less interesting conditions at the early stage, (ii) focused on DEGs that are related to muscle, and (iii) report the DEGs of interests while supporting the full list of DEGs that are related to muscle through their supplementary data. Therefore, to make both scenarios available in our study, we need to process the raw data of the tissues that are not directly supported in the original paper. Thus, we analyzed the raw RNA-seq data (GSE63268) with pipelines that are slightly different from the original paper. The pipeline we used reported 184 DEGS as up-regulated at HFD-ncls/HFD-veh while authors calculated them as 160. The details of RNA-seq data processing are explained in section ‘Materials’.

#### Authors’ approach : two DEG list

**Fig. 6:**
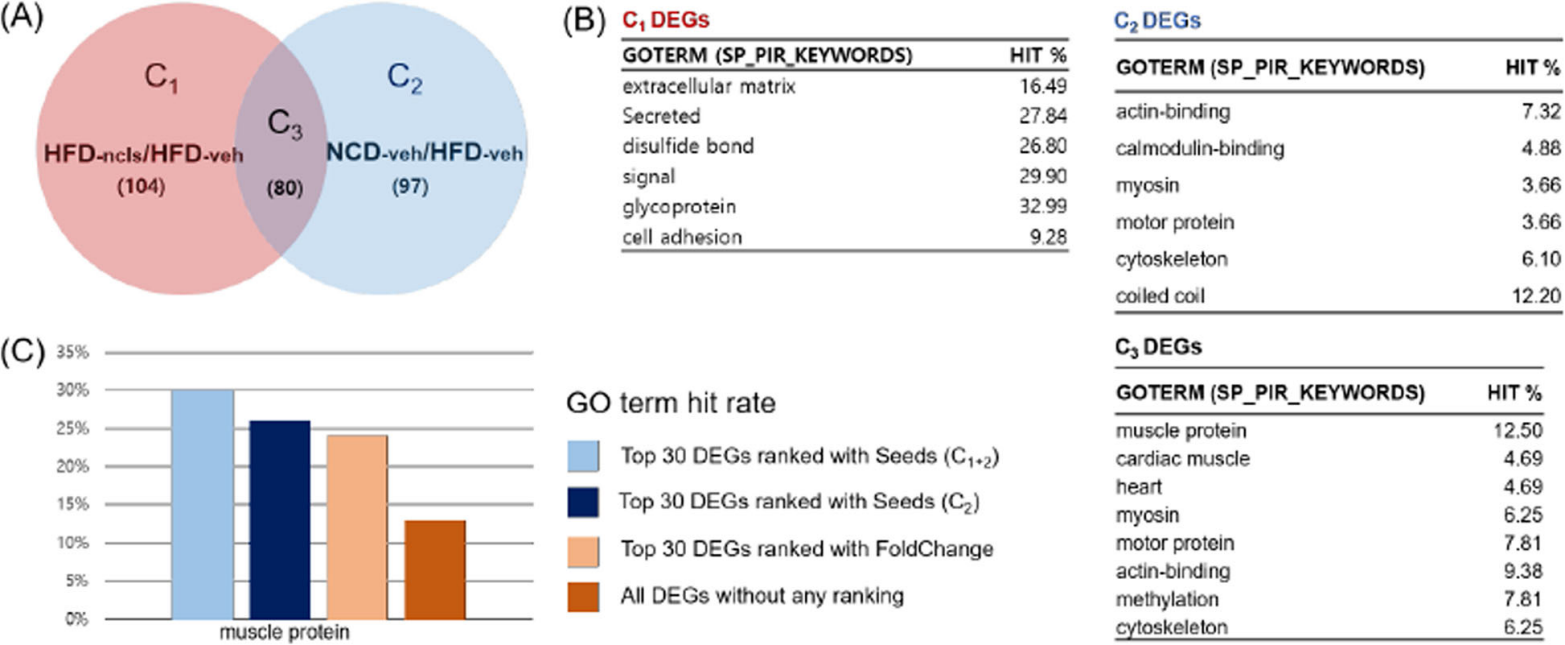
Venn-diaNet HFD GO term comparison. **a** Venn-diagram of GSE63268 experiment. *C*_1_ represents HFD (ncls/veh) specific DEGs while *C*_2_ shows veh (NCD/HFD) specific DEGs. **b** Enriched GO terms by DAVID gene functional clustering analysis. Gene functional clustering was performed for each specific region. **c** Enriched GO terms of Top 100 genes prioritized by corresponding seeds

As we described in the previous section, we assumed that we also performed hierarchical clustering and focus to find certain genes in C_3_ (Fig. 6A) that have the common characteristics of up-regulation when ncls is induced and up-regulated in NCD without any treatments.

In order to prioritize genes in C_3_, we used the seed scenario of Condition specific function as seeds. DEGs that are common in both experiments can be prioritized using the independent effects of each factor. Therefore, C_1_+C_2_, the specific effect of each treatments was selected as seeds to observe the influence to the genes that have same activity alteration in HFD-ncls/HFD-veh and NCD-veh/HFD-veh (C_3_). As a result, we found that Venn-diaNet could prioritize and reproduce the genes where the authors reported (Additional file 4:(2)) as well as prioritizing GO terms of the authors’ interest with better hit ratio (Fig. 6C). The minimum guideline, ‘Functional similarity as seeds’ (C_2_) showed weaker gene prioritization but still had a better focus on GO terms (Fig. 6C and Additional file 4:(2)). In addition, this study is designed to find the common effect from independent conditions, meaning that the condition of interest is closely related to each other condition. Therefore, it is natural to have poor performance with the same reason discussed in the previous section.

#### Venn-diaNet approach: All (eight) DEG list

**Fig. 7:**
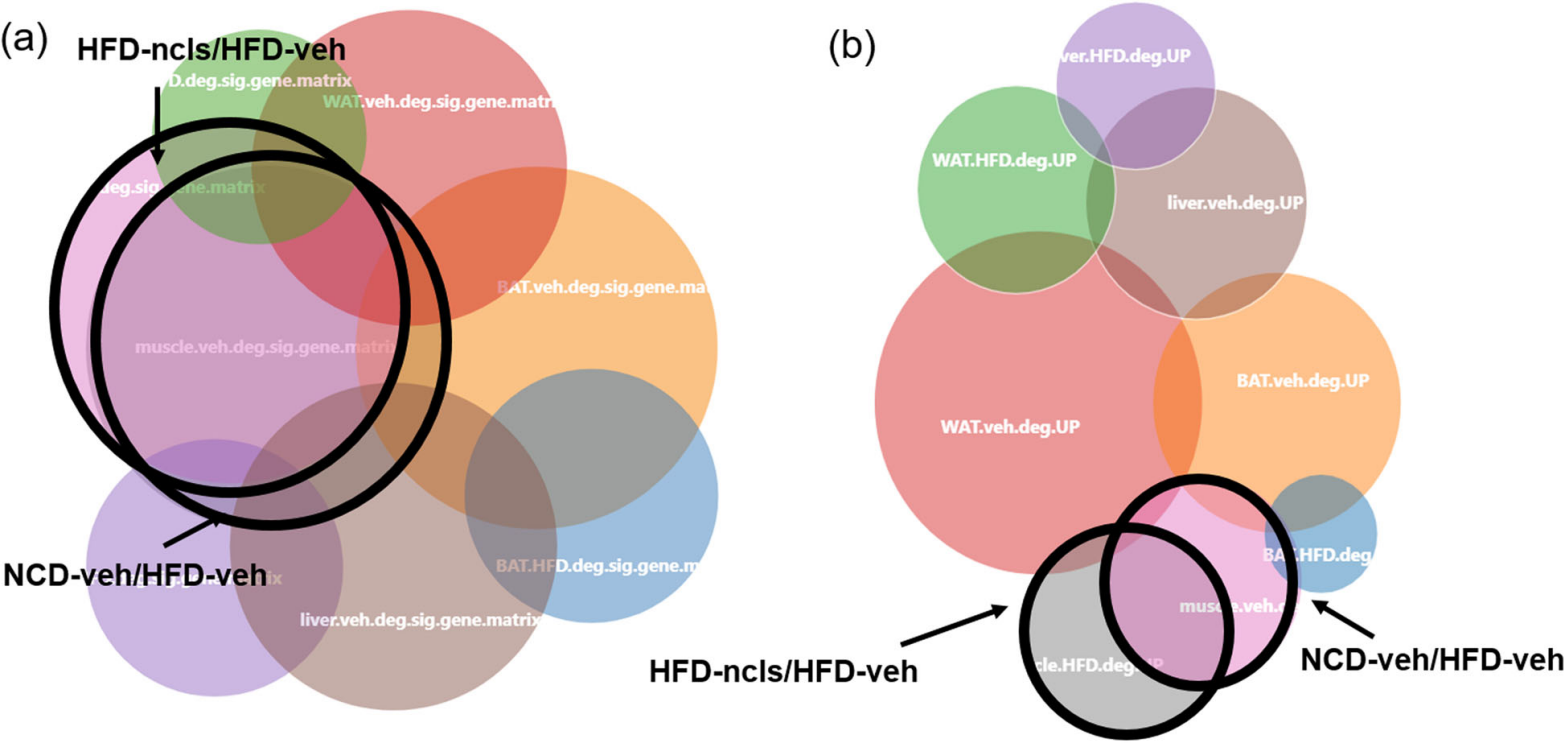
Venn-diaNet using 8 different DEG list. **a** Using up and down-regulated DEG list to Venn-diaNet (web). The Venn diagram directly shows muscle DEGs in HFD-ncls/HFD-veh, and NCD-veh/HFD-veh are similar to each other while other tissues are not similar to each other. **b** Using up-regulated DEG list to Venn-diaNet. The Venn diagram shows that up-regulated muscle DEGs in HFD-ncls/HFD-veh, and NCD-veh/HFD-veh are very similar to each other while other tissues are not similar to each other

We assumed ourselves that we do not have enough knowledge to this data, and tested whether Venn-diaNet could reach the same conclusion to the authors. We simply used Venn-diaNet with all DEG lists (that contains up and down-regulation) from eight different experiments at once (Fig. 7A). The Venn diagram shows that the intersection of HFD-ncls/HFD-veh and NCD-veh/HFD-veh shared many DEGs in muscle (*C*_48_) than any other tissues (*C*_3_, *C*_12_, *C*_192_).

The findings of Venn diagram reaffirms the authors’ hierarchical clustering results and leads to the idea that the intersection of HFD-ncls/HFD-veh and NCD-veh/HFD-veh in muscle have common functions than other tissues, and needs to be analyzed in detail. To start the detailed search, we now used up-regulated DEG list to examine whether Venn-diaNet can answer for the hypothesis of ‘ncls might accelerate genes to be expressed again while the genes were suppressed in HFD environment in muscle’. As a result, we were able to discover that the condition of interest was much more distinct to other conditions (Fig. 7B: *C*_48_) and the portion of common genes between HFD-ncls/HFD-veh and NCD-veh/HFD-veh in muscle was bigger than any other tissue (*C*_48_, *C*_3_, *C*_12_, *C*_192_). The findings indicate that up-regulation of *C*_48_ is likely to be more specific and distinct to other tissues. To prioritize genes in *C*_48_, we choose the seed scenario of ‘common functions as seeds’. We selected the intersection of HFD-ncls/HFD-veh and NCD-veh/HFD-veh of other tissues as seeds (*C*_3_, *C*_12_, *C*_192_) to represent that the function of ’ncls might accelerate genes to be expressed again while the genes are suppressed in HFD environment’ in other tissues can assist to prioritize genes in muscle. As a result, we were able to reproduce genes that the authors reported in their original paper (Additional file 4:(2)).

In addition to seed selection, the minimum guideline cannot be used for this complex condition data. The data is composed of 255 conditions that makes it difficult to compare and analyze the GO terms of all these conditions.

## System Architecture

Venn-diaNet is a web analysis tool built with Django web framework v.1.10.3 (https://djangoproject.com) and draws Venn diagram using venn.js [33]. venn.js draws keen Venn diagrams only with circles regardless to the number of conditions and considers the size of the circle and the position of the diagram’s centroid that depends on the size of the given sets. When the number of experiments is more than four, the drawn Venn diagram might not be perfectly correct, but it still considers the distance between circles as well as the size of circles to draw a reasonable Venn diagram as possible. d3.venngraph.js [34] is used to overlay the network graph upon the Venn diagram by using the position of each circle’s centroid. The distance between nodes was calculated by using the idea of the Nelder-Mead method to make nodes that have the same condition more closer while nodes that are distinct to the other conditions to be more further.

## Conclusions

We present Venn-diaNet, a web-based software that does not require any additional installment or registration. In this paper, we introduced that Venn-diaNet can be applied for various experiment designs and can effectively prioritize genes from multiple DEG lists. Experiment designs that have multiple lists of DEGs are generally difficult to prioritize phenotype related genes because it requires multiple data processes to gain a subset of gene lists. However, Venn-diaNet has shown that the combination of PPI network and Venn diagram can simplify these process. Venn-diaNet showed that the seeds from the segments of Venn diagram and the results of the network propagation with PPI network are effective enough to prioritize genes without considering the specific expression values of each DEGs lists. Also, because that Venn-diaNet can prioritize genes in a single step regardless to the number of DEGs lists, it has an advantage for analyzing complicated experiment designs that forces to analyze with multiple steps because it can simplify the analysis steps.

In addition, in the aspects of gene prioritization, Venn-diaNet can avoid the ‘black-box’ issue in gene prioritization which is caused by the integration of heterogeneous data sources because Venn-diaNet provides explainable ranking results of the network propagation [35]. Venn-diaNet supports gene list with ranking and additional features that explains how the specific gene is influential to other genes. Venn-diaNet is available at: (http://biohealth.snu.ac.kr/software/venndianet). Source code can be reviewed at: (https://github.com/hurben/VenndiaNet).

## Materials

### RNA-seq data processing

GSE63268, the dataset used in Case 3: Venn-diaNet for eight experiments, raw data (fastq) files were obtained from GEO [20], while RSEM (v1.2.19) and Bowtie2 (v2.2.6) were used for aligning reads. Reference genome (mm10) and gene annotation information was obtained from UCSC genome browser [36]. EBSeq [37], ‘rsem-run-ebseq’ was used for DEG calculation and 0.05 was used as a cutoff value for ‘rsem-control-fdr’. Every mentioned program was executed without any additional options. We would like to emphasize that the calculated DEGs slightly differs from the authors. In muscle, our pipeline calculated 184 DEGS as up-regulated at HFD-ncls/HFD-veh while authors calculated them as 160. We assume that the difference came from different reference genome and analysis pipeline which authors used mm9, and Cufflink. Despite the different pipeline and reference genome, key genes that the authors pointed out were still able to be reproduced. Details of gene list are in Additional file 4.

## Additional material

#### **Additional file 1**

A manual that describes the instructions of using Venn-diaNet (web)

#### **Additional file 2**

Supplementary data 1 is related to the detailed results of the experiment related to section Case 1: Venn-diaNet for two experiments

#### **Additional file 3**

Supplementary data 2 is related to the detailed results of the experiment related to section Case 2: Venn-diaNet for three experiments

#### **Additional file 4**

Supplementary data 3 is related to the detailed results of the experiment related to section Case 3: Venn-diaNet for eight experiments

## Abbreviations

BAT: Brown adipose tissue
DEG: Differentially expressed genes
GEO: Gene expression omnibus
GO: Gene ontology
HFD: High fat diet
HPV: Human papillomavirus
KO: Knockout
MRW: Markov random walk
NCD: Normal chow diet
PPI: Protein-protein interaction
WAT: White adipose tissue
WT: Wild type
